# Non-invasive MRI biomarkers for the early assessment of iron overload in a humanized mouse model of β-thalassemia

**DOI:** 10.1038/srep43439

**Published:** 2017-02-27

**Authors:** Laurence H. Jackson, Evangelia Vlachodimitropoulou, Panicos Shangaris, Thomas A. Roberts, Thomas M. Ryan, Adrienne E. Campbell-Washburn, Anna L. David, John B. Porter, Mark F. Lythgoe, Daniel J. Stuckey

**Affiliations:** 1 Centre for Advanced Biomedical Imaging, Division of Medicine, University College London, London, UK; 2 Department of Haematology, University College London, London, UK; 3 Institute for Women’s Health, University College London, London, UK; 4 Department of Biochemistry and Molecular Genetics, University of Alabama at Birmingham, Birmingham, Alabama, USA; 5 Laboratory of Imaging Technology, Biochemistry and Biophysics Center, National Heart, Lung, and Blood Institute, National Institutes of Health, Bethesda MD, USA

## Abstract

β-thalassemia (βT) is a genetic blood disorder causing profound and life threatening anemia. Current clinical management of βT is a lifelong dependence on regular blood transfusions, a consequence of which is systemic iron overload leading to acute heart failure. Recent developments in gene and chelation therapy give hope of better prognosis for patients, but successful translation to clinical practice is hindered by the lack of thorough preclinical testing using representative animal models and clinically relevant quantitative biomarkers. Here we demonstrate a quantitative and non-invasive preclinical Magnetic Resonance Imaging (MRI) platform for the assessment of βT in the γβ^0^/γβ^A^ humanized mouse model of βT. Changes in the quantitative MRI relaxation times as well as severe splenomegaly were observed in the heart, liver and spleen in βT. These data showed high sensitivity to iron overload and a strong relationship between quantitative MRI relaxation times and hepatic iron content. Importantly these changes preceded the onset of iron overload cardiomyopathy, providing an early biomarker of disease progression. This work demonstrates that multiparametric MRI is a powerful tool for the assessment of preclinical βT, providing sensitive and quantitative monitoring of tissue iron sequestration and cardiac dysfunction- parameters essential for the preclinical development of new therapeutics.

β-thalassemia is an inherited blood disorder that causes the production of abnormal and ineffective red blood cells leading to profound and life threatening anemia. β-thalassemia patients present severely reduced synthesis of the beta globin chain component of adult hemoglobin, the resulting α- and β-globin chain imbalance leads to ineffective erythropoiesis^1^,^2^. The most severe form of this condition is β-thalassemia major (βTM) in which β-globin production is totally absent. Current clinical management of disease is a lifelong dependence on regular blood transfusions, a consequence of which is systematic cardiac iron overload leading to acute heart failure. At birth βT patients survive on fetal hemoglobin (HbF) and do not present symptoms until completing the transition to adult hemoglobin (HbA) at around 6 months of age. Survival into adulthood is then dependent on regular blood transfusions^3^,^4^. A consequence of repeated transfusions is the accumulation of iron due to the lack of any innate iron excretion mechanism and pathological transferrin saturation. Transfusion induced iron accumulation is compounded by increased gastrointestinal iron absorption as a result of hepcidin suppression associated with ineffective erythropoiesis^5^. If left untreated, iron accumulation leads to tissue iron overload and acute heart failure as a result of cardiac siderosis. Myocardial iron removal is slow with existing chelation regimens and the development of more effective therapies and curative approaches is limited by the paucity of relevant animal models in which testing of new treatments can be investigated^6^,^7^,^8^.

Mouse models for βT have been produced by deletion of the mouse β-globin genes, however in the absence of β-globin expression these mice die in utero making them unsuitable for serial studies^2^,^9^,^10^,^11^,^12^. Since mice have no equivalent of human HbF, these models do not express the characteristic transition from HbF to HbA seen in humans. Recent work by Huo *et al*. have developed humanized mouse models of βT that closely mimic the temporal fetal-to-adult hemoglobin switch and onset of anemia seen in humans with βT^13^,^14^,^15^,^16^. With the advent of representative animal models there is an urgent need for accurate non-invasive methods able to detect and quantify iron load so that new therapies can be directly assessed *in vivo*.

The use of clinical magnetic resonance imaging (MRI) to assess the extent of iron loading in organs has revolutionized the diagnosis, management and treatment of βT patients^17^,^18^,^19^,^20^. MRI has two primary advantages over other imaging or invasive diagnostic techniques; accurate measurement of cardiac structure and function; and direct quantification of iron loading due to shortening of magnetic relaxation times T1, T2 and T2* in the presence of iron. These properties make it the ideal tool for clinical monitoring of βT patients providing noninvasive monitoring of cardiac iron deposition and the consequential iron induced cardiomyopathy. Although MRI in thalassemia has become clinical practice where available, translation of quantitative MRI techniques to preclinical mouse models is challenging. Challenges to quantitative MRI include the rapid murine heart rate, approximately 600bpm and small myocardium that is typically just 1mm thick. The consequence is that *in vivo* murine studies implementing quantitative relaxometry of T2, T2* and T1 values in the myocardium have been limited especially in the case of T2 and T2* measurements that are more susceptible to cardiac motion^21^,^22^,^23^,^24^,^25^,^26^.

This study developed clinically relevant MRI assessment methods to the novel humanized γβ^0^ knockin mouse model of β-thalassemia in the presence of iron overload, for the first time. Tissue magnetic resonance relaxation rates (T1/T2/T2*), spleen volumetrics and cardiac function were quantified and demonstrates the utility of preclinical MRI assessment in small animal models of thalassemia as an enabler to *in-vivo* and non-invasive serial investigation for the development of new therapies.

## Materials and Methods

### Animal models

All animal studies were approved by the University College London Biological Services Ethical Review Committee and licensed under the UK Home Office regulations and the Guidance for the Operation of Animals (Scientific Procedures) Act 1986 (Home Office, London, United Kingdom). All animal methods were performed in accordance to institutional ethical guidelines and regulations. The mouse models of βT were created by replacing the adult mouse α- and β-globin genes with human α-globin and human γ- to β-globin hemoglobin switching cassette (γβ^0^ or γβ^A^). Homozygous fully humanized knockin (γβ^0^/γβ^0^) mice survive to birth by synthesizing solely human HbF and are an accurate model of βTM. Heterozygous fully humanized knockin (γβ^0^/γβ^A^) mice show typical symptoms of β-thalassemia following completion of the fetal-to-adult hemoglobin switch including splenomegaly and mild anemia despite considerable changes in red blood cell (RBC) indices. Mice homozygous for the γβ^A^ knockin (γβ^A^/γβ^A^) survived into adulthood by synthesizing human HbA, this model acted as a wild type control to account for the presence of the human globin genes. Three groups of animals were studied:


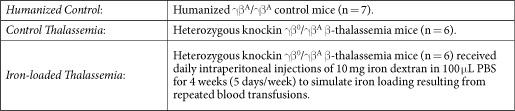


Humanized control and control thalassemia mice received injections of PBS with the same regimen. All injections began at 4 Months of age.

### *In vivo* MRI

Imaging was performed at 5 months of age using a 9.4 T MRI system (Agilent Technologies, Santa Clara, USA) equipped with 1000mT/m gradient inserts and a 39mm volume resonator RF coil (RAPID Biomedical, Rimpar, Germany). A small animal physiological monitoring system (SA Instruments, Stony Brook, NY) was used to maintain depth of anesthesia and animal physiology. The ECG trace was recorded using 3-lead subcutaneous electrodes, respiration rate was measured by a pressure sensitive balloon and internal temperature by rectal thermometer. Animals were anesthetized under a mixture of 1–2% isoflurane in oxygen.

### Spleen volume

Spleen volume data were acquired in control and non-loaded thalassemia mice using a respiration gated multislice gradient echo axial sequence (GEMS) with in-plane resolution 156 μm/px, slice thickness 0.5 mm, flip angle 20°, TE 3.2 ms. In iron loaded mice due to ultra-short T2/T2* relaxation in the spleen a GEMS protocol was impractical, in this case a respiration gated T1-weighted spin-echo multislice sequence was used with in-plane resolution 234 μm, slice thickness 1 mm, TE 2.5 ms, TR 600 ms. In both cases the number of slices was adapted to the cover the whole spleen volume. Image data was reconstructed offline and spleen tissue was identified by drawing organ contours on individual 2D slices and propagating through plane to measure organ volume. The ratio of spleen volume to total animal mass was used as a quantitative and animal-independent value for spleen size.

### Relaxometry

Transverse relaxation mechanisms T2 and T2* are the time constants describing the rate of transverse MRI signal decay and depend on the microscopic magnetic environment of tissue. The large paramagnetic influence of iron storage molecules ferritin and hemosiderin cause perturbations to the magnetic environment. This results in faster transverse relaxation proportional to the concentration iron species, making T2/T2* quantifiable markers for iron-overload^27^,^28^.

T2 relaxation was measured using a cardiac and respiratory gated spin echo sequence where a respiratory gate triggered acquisition of one phase encoding line per slice per R-wave until the next respiration. This acquisition was repeated at 8 echo times ranging from 2.7–20 ms. Typical respiration and heart rates allowed for 7 slices with 0.3 × 0.3 × 1.5mm/px resolution covering 6 slices in the heart and liver with one through the spleen. The T2 signal relaxation could then be fitted to a model of transverse signal decay: 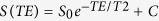 where *TE* is echo time, *S*_0_ is the initial signal intensity and *C* is a fitted offset to account for image noise.

T2* relaxation was then measured using two multi gradient echo sequences with 15 echo times in the range of 0.9–14.9 ms at 1ms intervals. These images had a 0.23 × 0.23 × 1.5 mm/px resolution. One slice was placed in the same orientation as the T1 look locker and second acquisition was orientated in the spleen. The signal intensities were then modelled as T2* relaxation using according to the same model as T2 with T2* substituted for T2. Both T2 and T2* are shortened in the presence of iron, however T2* can be effected by other magnetic susceptibility induced field changes in particular the effect of the air filled lung cavity can artificially shorten T2* in the heart. To limit this effect all cardiac T2* measurements used only the intraseptal region where the magnetic field is most uniform.

To measure T1 relaxation a cardiac gated look locker sequence was used with a minimum TE of 2.8 ms followed by 30 inversion times separated by the RR interval^29^. The slice was orientated so as to cover the mid-papillary level short axis of the heart and a portion of liver allowing for both tissues to be quantified during a single scan with an in-plane resolution of 0.3 mm/px and a slice thickness of 1.5 mm. This resulted in an image sequence where signal intensity could be modelled by a 3 component model 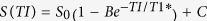, where TI is the time following the radiofrequency look locker inversion pulse, *B* is a fitted parameter to account for imperfect inversion and *T*1* is the apparent T1 under the influence of look locker saturation. This is corrected by the correction factor 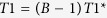. Due to the long sampling time for T1 decay the acquisition was acquired over respirations, images corrupted with respiration motion were automatically excluded from fitting using the phase-encoded noise-based image rejection scheme^30^.

Tissue regions were segmented and models fitted to mean ROI values at each echo time. Model fitting was performed using in house Matlab optimization code (2014b, The Mathworks, Inc., Natick, USA) based on the Nelder-Mead Simplex Method.

### Cardiac function

Cardiac function was assessed with a spoiled gradient echo cine MRI sequence with a temporal resolution of 5 ms where the number of cine frames was matched to cover the complete cardiac cycle. The acquisition used an in plane spatial resolution of 117 μm/px and a slice thickness of 1 mm. The left ventricular blood pool was segmented at systole and diastole using Segment v1.8 R0462^31^ and the corresponding volumes used to calculate left ventricular ejection fraction (EF), stroke volume (SV) and end systolic/diastolic volumes (ESV/EDV)^32^.

### Iron quantification and histology

Following imaging animals were sacrificed and samples of heart, spleen and liver were fixed in 4% PFA for analysis. Non-heme tissue iron concentration was measured using the iron assay described by Bothwell *et al*.^33^ and iron deposits were observed histologically by Perls’ stain.

### Statistical tests

All results are presented as mean value ± standard error. All data were tested for normality using the Kolmogorov-Smirnov test and significance values were calculated by one-way analysis of variance corrected for multiple comparisons using the Holm-Šídák method. In all cases a p-value of less than 0.05 was considered significant.

## Results

### T2/T2* relaxation

**Figure 1:**
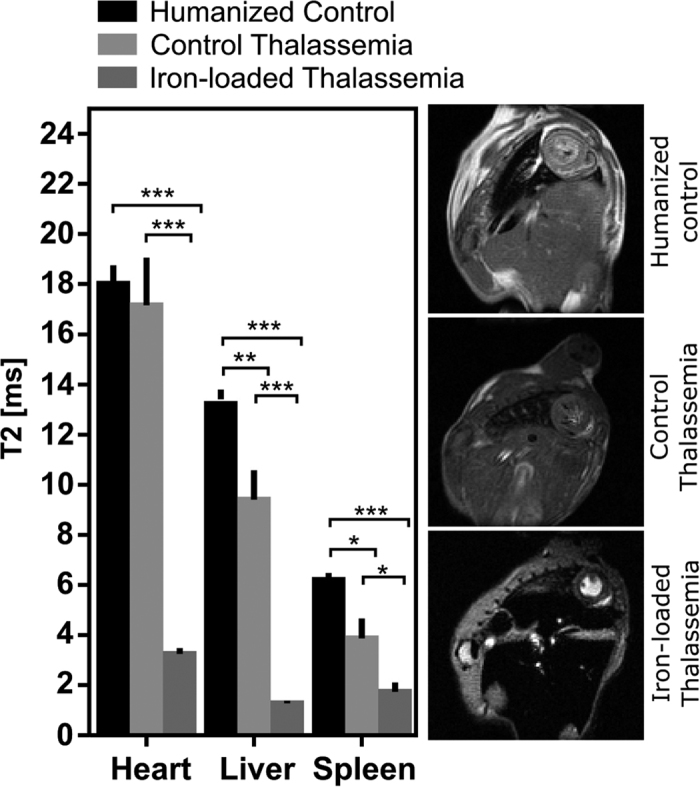
T2 relaxation times in the heart, liver and spleen with representative images at TE = 5 ms. T2 was shortened in the liver and spleen for the control thalassemia animals and more severely shortened in the case of iron-loaded thalassemia mice. Cardiac T2 was shortened in the iron loaded thalassemia mice but not the control thalassemia animals. Representative images depict oblique slices showing the cardiac short axis and liver, in iron loaded animals the liver shows severe signal hypointesity and there is a visibly progressing drop in signal between images matching the T2 measurements. (*p < 0.05, **p < 0.01, ***p < 0.001).

**Figure 2:**
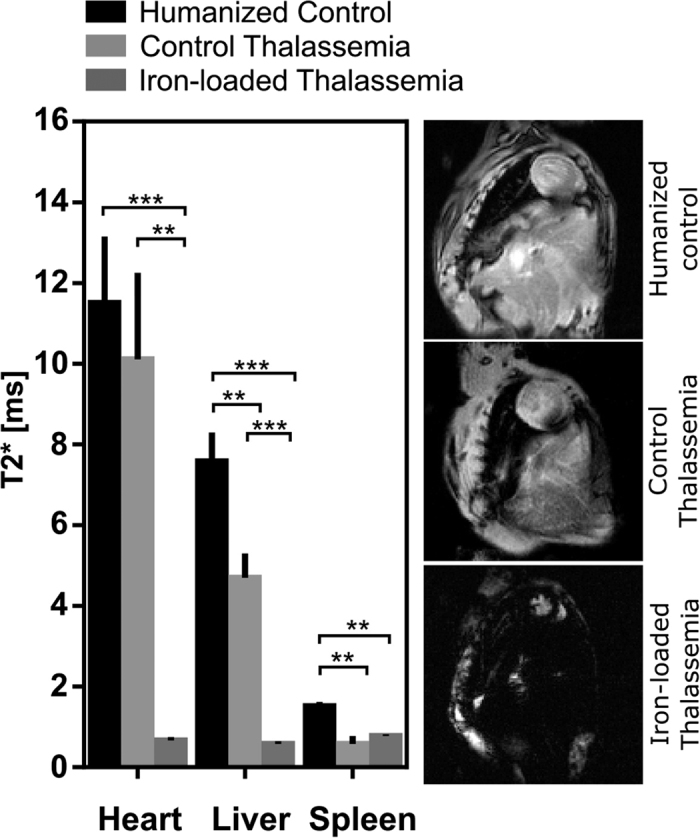
T2* relaxation times in the heart, liver and spleen with representative images at TE = 1.8ms. T2* relaxation was shortest in the iron-loaded thalassemia animals in the heart, spleen and liver. The hepatic T2* of the control thalassemia mice also showed a significant (p = 0.0042) reduction. Cardiac T2* showed the same trend as T2 with no significant change between control and thalassemia controls. (*p < 0.05, **p < 0.01, ***p < 0.001).

Measurements of cardiac, hepatic and splenic T2 and T2* are shown in Fig. 1 and Fig. 2 respectively, along with representative images from each group at identical echo times for comparison.

Myocardial T2 was greatly shortened in iron-loaded thalassemia mice (3.3 ± 0.3 ms) versus humanized controls (18.0 ± 0.8 ms) and thalassemia controls (17.2 ± 2.1 ms). Similarly, myocardial T2* in iron overload (0.7 ± 0.2 ms) was shortened relative to humanized controls (11.5 ± 4.3 ms) and thalassemia controls (10.1 ± 5.2 ms). These data show that T2 and T2* show detectable sensitivity to iron loading in this animal model. There was no significant difference in T2 or T2* between the non-iron loaded groups indicating that the additional iron uptake in the heterozygous humanized γβ^0^/γβ^A^ model is not severe enough in itself to produce a detectable change in cardiac T2 or T2* at 5 months of age.

Hepatic T2 was shortened in both the control thalassemia (9.4 ± 2.9 ms) and iron-loaded thalassemia mice (1.3 ± 0.3 ms) relative to humanized controls (13.2 ± 01.0 ms). Similarly, hepatic T2* was shortened in the control thalassemia (4.7 ± 1.5 ms) and iron-loaded thalassemia mice (0.6 ± 0.2 ms) models relative to humanized controls (7.6 ± 1.9 ms). This shortening of T2 and T2* in both the thalassemia groups can be related to the role of the liver as the primary reserve for body iron stored as ferritin. In the thalassemia control group increased dietary iron uptake could explain this shortening while in the iron-loaded thalassemia group we see this effect exaggerated by the additional exogenous iron deposition.

The role of the spleen in breaking down senescent erythrocytes makes it hyperactive in thalassemia where a large population of erythrocytes are defective. Splenic iron deposition is primarily due to the break-down of erythrocytes and hemoglobin in the spleen. The heme by-product is transported to the liver by transferrin which in thalassemia can become saturated preventing removal. The shortened T2 and T2* in both thalassemia groups reflects this process. Splenic T2 was shortened in control thalassemia mice (3.9 ± 0.8 ms) and iron-loaded thalassemia (1.7 ± 0.9 ms) compared with humanized controls (6.2 ± 0.8 ms). Splenic T2* was further shortened in iron overload mice (0.8 ± 0.1 ms) and control thalassemia (0.6 ± 0.5 ms) relative to humanized controls (1.5 ± 0.3 ms).

It is known that iron accumulation occurs initially in the macrophage system of the spleen, liver and bone marrow but subsequently in hepatocytes and ultimately in the heart and endocrine systems. These MRI measurements show that at 5 months of age the liver and spleen show signs of iron loading but the process has not yet begun to accumulate iron in the heart suggesting that this is a relatively early stage of disease.

### T1 relaxation

**Figure 3:**
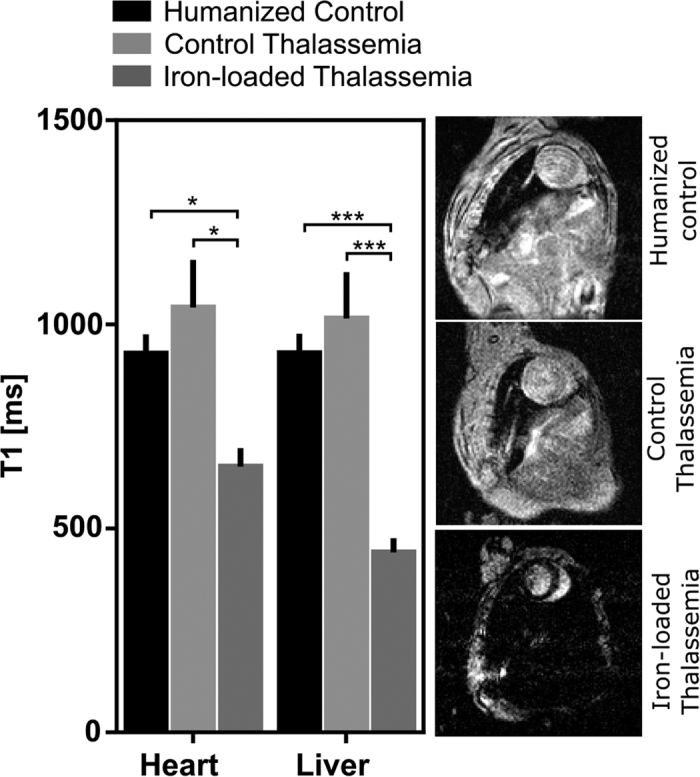
T1 relaxation times in the heart, liver and spleen with representative images at TI = 110ms. T1 relaxation in heart and liver was significantly shortened relative to controls in iron-loaded thalassemia mice (p = 0.0455 and p = 0.0009 respectively). In both the liver and spleen but not significantly effected in the thalassemia control mice. (*p < 0.05, ***p < 0.001).

T1 values were significantly shorter in the presence of iron loading (Fig. 3). Cardiac T1 was shorter in iron-loaded mice (620 ± 125 ms) relative to control thalassemia mice (1041 ± 261 ms) and humanized control animals (928 ± 115 ms). This was also the case for hepatic T1, with iron loaded thalassemia animals having shorter T1 (456 ± 85 ms) than control thalassemia (1014 ± 255 ms) and humanized control animals (929 ± 117 ms). These data show that T1 is sensitive to the presence of iron loading however, T1 changes in the presence of iron require direct interaction with ferritin or hemosiderin, the sensitivity is therefore lower than T2/T2*. T1 measurements could therefore be useful at particularly high iron concentrations where T2/T2* can be too short to accurately quantify.

### Spleen volume

**Figure 4:**
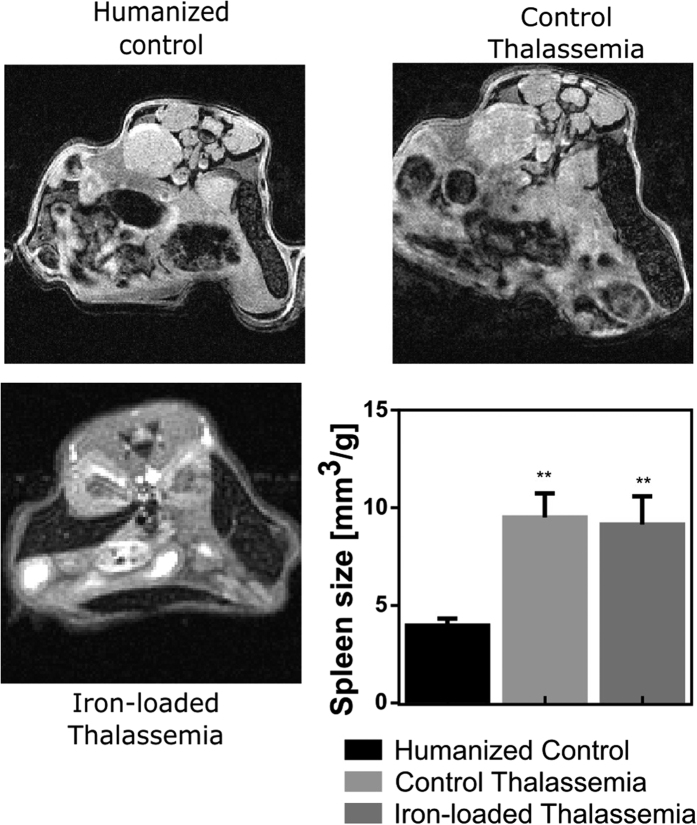
Representative images showing the enlargement of the spleen in thalassemia. Spleens (S) were dramatically enlarged relative to humanized controls in control and iron-loaded thalassemia mice. The spleen can be identified in MRI images by its speckled texture due to the dense trabeculae. Spleen volume was quantified by the ratio of spleen volume to animal mass. (**p < 0.01).

A severe increase in spleen size was observed in the presence of the γβ^0^ gene. Figure 4 shows a comparison of the axial spleen images and highlights the enlarged spleens in iron loaded and control thalassemia mice. Spleen volume normalized to body mass was significantly increased in control thalassemia (9.5 ± 1.2 mm^3^/g) and iron loaded thalassemia mice (9.1 ± 1.3 mm^3^/g) relative to humanized control mice (4.0 ± 0.4 mm^3^/g), however there was no significant change in volume between iron loaded and control thalassemia mice suggesting that splenomegaly in this animal model is a purely a consequence of γβ^0^ knockin and is not directly affected by the degree of iron deposition.

### Cardiac function

**Figure 5:**
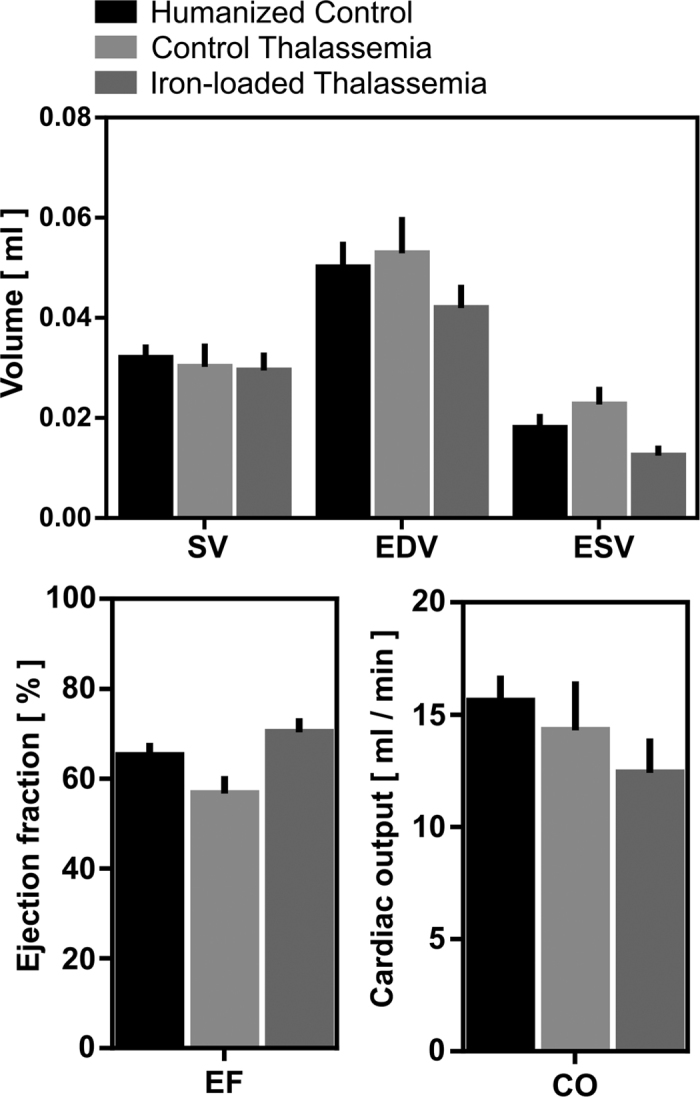
Measurements of myocardial function from cine MRI. No significant changes in cardiac volumes were observed between groups, although the iron-loaded thalassemia animals had an elevated ejection fraction relative to control thalassemia mice. This suggests that iron induced cardiomyopathy has not yet developed suggesting MRI relaxometry could be a useful early biomarker for the onset of βT.

Quantitative values describing left ventricular function are shown in Fig. 5. Left ventricular end diastolic, end systolic and stroke volumes (SV) were preserved between groups. Cardiac output (CO), measured as the product of stroke volume and heart rate was not significantly altered in control thalassemia mice (14.1 ± 2.1 ml/min) relative to humanized controls (15.6 ± 1.1 ml/min) and iron-loaded mice (12.4 ± 1.5 ml/min) indicating that at 5 months of age that the two heterozygous knockin groups have not developed the pathological high output state characteristic of anemia. Left ventricular EF was also unchanged in iron-loaded mice (70 ± 2%) relative to control thalassemia mice (57 ± 4%) and humanized control animals (65 ± 2%).

### Histology

**Figure 6:**
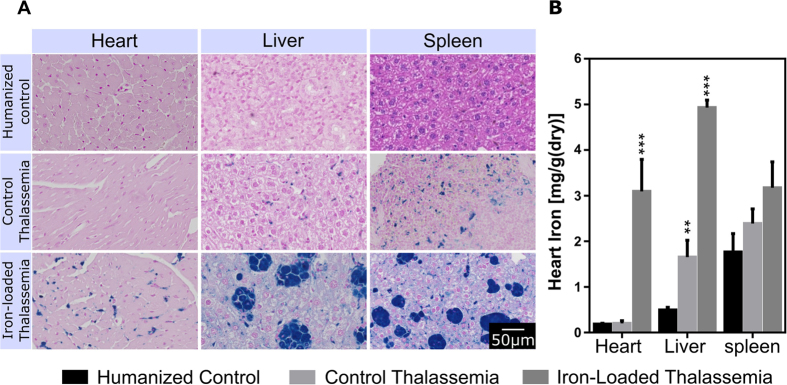
Histological validation of iron concentrations in the heart, liver and spleen. Perls stain for iron (**A**) show iron deposits in the spleen, liver and heart. Iron accumulation in the heart only occurs following iron loading and not in control thalassemia mice. Results of the Bothwell iron assay (**B**) show that T2 and T2* measurements are good markers for iron content, significance relative to humanized controls (*p < 0.05, ***p < 0.001). Iron content shows an inverse relationship with transverse relaxation mechanisms in the liver.

Histological sections of spleen, heart and liver are shown in Fig. 6A. Small iron deposits are visible by blue/purple staining in the liver and spleen of the thalassemia control group but there are no iron deposits visible in cardiomyocytes. This iron distribution is consistent with early stage iron overload where the iron has not spread beyond the macrophage system in the liver and spleen. In the iron loaded thalassemia group there are large iron deposits in the liver and spleen but crucially iron deposits are now visible in the myocardium. Iron concentration determined by the Bothwell non-heme assay for each organ and group is shown in Fig. 6B. Control thalassemia hearts did not show significantly higher iron content than humanized controls but iron loaded thalassemia animals showed large myocardial iron depositions. Hepatic iron content was higher than humanized controls in control thalassemia and iron-loaded animals while splenic iron showed an increasing trend but the results did not reach statistical significance. These results match the T2/T2* relaxometry measurements verifying these data.

### Iron calibration

**Figure 7:**
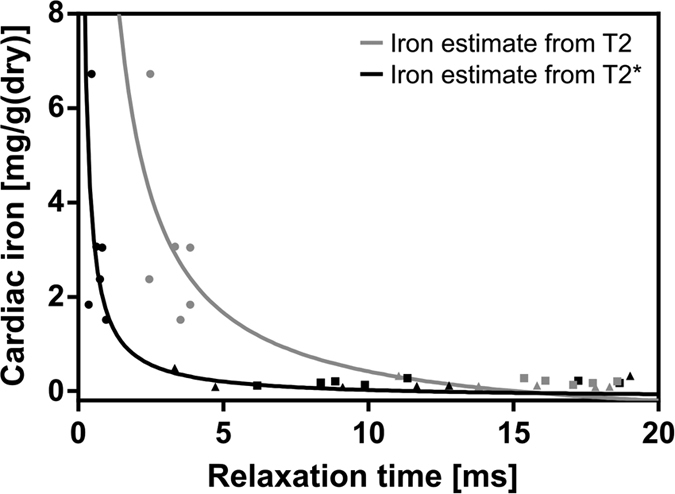
Calibration curves for myocardial dry iron concentration from cardiac T2 and T2* relaxation times. Creating a calibration curve between these two measurements allows estimations of cardiac iron content to be made without the use of an invasive cardiac iron assay. Points show individual animals, humanized controls (■), Thalassemia controls (▲) and iron-loaded thalassemia (•), grey/black points correspond to the T2/T2* measurements respectively.

The cardiac T2* and iron dry weight measurements can be used to create a calibration curve to estimate iron content of dry tissue. This relationship can be modeled as 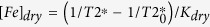 where T2* is the measured T2* value, T2_0_* is the T2* of the heart in the absence of iron loading and K_dry_ is a proportionality constant for dry tissue^24^. Here we take the mean T2* of humanized controls as T2_0_* and fitted K_dry_ to the measured T2* and dry iron concentrations. The resulting calibration curve fits measured values well (Fig. 7) and shows a sharp increase in estimated tissue iron at T2* < 2 ms. The process can be repeated for T2 measurements by substituting T2 for T2*. In this case we find that iron concentration increases sharply at T2 < 5 ms.

## Discussion

In humans T2* MRI is the primary technique for identifying pathological iron overload in βT and has revolutionized the early diagnosis of disease prompting leaps in life expectancy as MRI becomes more readily available^1^. A myocardial T2* of <10 ms is the recognized primary predictor of iron induced heart failure in patients with iron overload. In this study we find a similar rapid increase in estimated cardiac iron content at <2 ms. The T2 and T2* calibration curves fitted in Fig. 7 are representative of calibration curves seen in humans. This similar pattern is a promising sign that the animal model is clinically representative and that serial measurements of tissue iron by MRI can establish trends in tissue iron loading during the onset and following the treatment of βT, enabling more clinically useful interpretation of efficacy of such treatments.

The small organ size of rodents necessitates the use of high field MR imaging systems to obtain adequate temporal and spatial resolution and brings major challenges to imaging due to shortened relaxation times and accentuated field inhomogeneity. T2* is currently the clinical standard for assessment of iron overload and has been universally adopted, however T2* is influenced by a number of factors independent of tissue iron concentration. The dense capillary networks within the myocardium have a significant impact on cardiac T2* relaxation due to their tortuous geometry and magnetic susceptibility which can be altered in disease. In βT blood of varying degrees of oxygenation can drastically alter relaxation rates due to the different magnetic properties of oxy and deoxyhemoglobin. Variation in B0 shimming and long range susceptibility effects from the lungs can also influence measurements. Alternatively, T2 relaxation is less affected by these variations but has a lower sensitivity to iron. Here we found that T2 and T2* provide the same information with regards to iron concentration in the heart, liver and spleen. It is therefore feasible to use T2 as a more accurate quantitative measure of tissue iron when high sensitivity to iron is not required. T2 has also been shown to be a useful and reproducible measure of tissue iron in humans, however T2* remains the gold standard due to the substantial number of validation studies^34^,^35^.

The true relationship between myocardial iron and T2/T2* relaxation has previously proven difficult to determine. Although the shortening effect of iron is recognized it must be determined that iron is the dominant factor in faster transverse relaxation in βT as opposed to the abnormal hemodynamics. Iron concentration in this study was found to follow the same trend as T2 and T2* values for each organ. The control thalassemia and iron-loaded thalassemia groups have identical γβ^0^/γβ^A^ knockin genes and present the same variable blood oxygenation and myocardial capillary networks of βT. The iron-loaded animals demonstrated a shortening of T2 and T2* regardless of this providing evidence that the dominant T2/T2* shortening factor in βT is iron accumulation.

Pronounced splenomegaly was observed in the γβ^0^ knockin mice irrespective of the presence of iron overload in the organ. This is consistent with the main cause of splenomegaly in βT being the extended tissue hypoxia with splenic siderosis playing a minor role in organ dysfunction. Since the spleen functions primarily as a filter to remove damaged RBCs it can become hyperactive in the case of βT due to the high population of defective RBCs^36^,^37^. Hypersplenism and splenomegaly shorten the lifetime of transfused RBCs creating a requirement for more frequent transfusions and compounding the consequential iron overload, therefore successful treatment of βT will require slowing or prevention of hypersplenism. In this study MRI has been shown to be a useful tool for assessing the extent of organ enlargement and the ability to serially measure this *in vivo* will be valuable for therapeutic development.

In anemic states the heart compensates for low blood oxygen content by increasing cardiac output to maintain tissue oxygenation, typically through increasing end diastolic volume^38^. Results here however do not show a significant increase of these cardiac metrics in γβ^0^/γβ^A^ knockin mice relative to humanized γβ^A^/γβ^A^ controls. The likely explanation for this is the relatively mild anemia in the heterozygous γβ^0^/γβ^A^ knockin mice, meaning that compensatory increases in cardiac output are not required. Although cardiac dysfunction is not observed, an increase in ferritin and hemosiderin from assay and relaxometry measurements is seen in iron loaded and thalassemia mice and is known to predispose a patient to cardiac dysfunction in the future due to the corresponding increase in toxic labile iron^39^. The onset of cardiac dysfunction in the presence of iron overload is well established and the data presented here suggests that relaxometry measurements and increased spleen volume precede the onset of cardiac dysfunction and can be used as early markers for the progression of disease and is the same in the clinical setting^1^.

Current research into therapies for βT typically focuses on either gene therapy, novel iron chelation agents or inducing post-natal production of HbF. Gene therapy approaches aim to modify harvested autologous hemopoietic stem cells *in-vitro*, repairing the dysfunctional β-globin genes before transplanting them back to the patient^40^. Gene therapy has been shown to be effective in mice for various forms of β-thalassemia^41^,^42^,^43^,^44^. These studies required animals to be culled at each time point to assess severity of disease, the *in-vivo* nature of MRI imaging means it would be a valuable addition to these studies providing a means to characterizing the time course of treatment. Iron chelation therapy is a necessary accompaniment to regular blood transfusions. Currently there are just three common clinically approved iron chelators: Deferoxamine, Deferiprone and Deferasirox. Each drug has its relative adverse effects and challenges meaning that the development of new agents is an active field of research^45^. There are promising agents such as FBS0701 that has many advantageous properties relative to current chelators and is currently undergoing phase II clinical trials in which MRI assessment of iron concentration and cardiac function are primary endpoints^46^. FBS0701 has undergone preclinical assessment of toxicity and safety but these studies did not utilize MRI techniques which would have provided translatable information for clinical trials^47^,^48^. Future preclinical studies into the safety of new agents will greatly benefit from integrating the MRI assessments that we present here, allowing parallel investigation of toxicity and iron chelation efficacy serially *in vivo*.

The data presented here demonstrates that MRI can be used to characterize the γβ^0^ knockin mouse model of βT. Both spleen volumetrics and relaxometry distinguished between humanized controls, control thalassemia knockin mice and iron-loaded thalassemia mice with a marked increase in spleen volume and shortening of relaxation times in thalassemia mice. The increasing iron content in the thalassemia animals quantified by chemical iron assay and visualized by Perls’ stain (Fig. 6) confirms the inverse relationship between increasing iron load and decreased T2 and T2* relaxation time in tissue.

## Conclusion

In this work we have presented the first study to use multiparametric quantitative MRI to assess tissue iron, spleen volume and cardiac function in the γβ^0^ knockin mouse model of β-thalassemia. Measurements of T2/T2* show great potential as a specific and sensitive biomarker for preclinical monitoring of iron accumulation, while measurements of spleen volume and heart function are able to provide additional biomarkers to assess disease progression. Using MRI to quantify βT in experimental animal models will be a powerful tool to assist in the development of new therapies and is directly translatable to clinical practice.

## Additional Information

**How to cite this article:** Jackson, L. H. *et al*. Non-invasive MRI biomarkers for the early assessment of iron overload in a humanized mouse model of β-thalassemia. *Sci. Rep.* **7**, 43439; doi: 10.1038/srep43439 (2017).

**Publisher's note:** Springer Nature remains neutral with regard to jurisdictional claims in published maps and institutional affiliations.
